# Annexin A1_2–26_ Treatment Improves Skin Heterologous Transplantation by Modulating Inflammation and Angiogenesis Processes

**DOI:** 10.3389/fphar.2018.01015

**Published:** 2018-09-10

**Authors:** Jéssica Zani Lacerda, Carine Cristiane Drewes, Kallyne Kioko Oliveira Mimura, Caroline de Freitas Zanon, Tahera Ansari, Cristiane Damas Gil, Karin Vicente Greco, Sandra Helena Poliselli Farsky, Sonia Maria Oliani

**Affiliations:** ^1^São Paulo State University (Unesp), Institute of Biosciences, Humanities and Exact Sciences (Ibilce), São Paulo, Brazil; ^2^Department of Clinical and Toxicological Analysis, Faculty of Pharmaceutical Sciences, University of São Paulo, São Paulo, Brazil; ^3^From the Post-Graduation in Structural and Functional Biology, Federal University of São Paulo, São Paulo, Brazil; ^4^Department of Surgical Research, Northwick Park Institute for Medical Research, University College London, London, United Kingdom

**Keywords:** Ac2–26 peptide, dorsal skinfold chamber, HUVEC, VEGF-A, cytokines, fibroblast

## Abstract

Skin graft successful depends on reduction of local inflammation evoked by the surgical lesion and efficient neovascularization to nutrition the graft. It has been shown that N-terminal portion of the Annexin A1 protein (AnxA1) with its anti-inflammatory properties induces epithelial mucosa repair and presents potential therapeutic approaches. The role of AnxA1 on wound healing has not been explored and we investigated in this study the effect of the peptide Ac2–26 (N-terminal AnxA1 peptide Ac2–26; AnxA1_2–26_) on heterologous skin scaffolds transplantation in BALB/c mice, focusing on inflammation and angiogenesis. Treatment with AnxA1_2–26_, once a day, from day 3–60 after scaffold implantation improved the take of the implant, induced vessels formation, enhanced gene and protein levels of the vascular growth factor-A (VEGF-A) and fibroblast influx into allograft tissue. It also decreased pro- while increasing anti-inflammatory cytokines. The pro-angiogenic activity of AnxA1_2–26_ was corroborated by topical application of AnxA1_2–26_ on the subcutaneous tissue of mice. Moreover, treatment of human umbilical endothelial cells (HUVECs) with AnxA1_2–26_ improved proliferation, shortened cycle, increased migration and actin polymerization similarly to those evoked by VEGF-A. The peptide treatment instead only potentiated the tube formation induced by VEGF-A. Collectively, our data showed that AnxA1_2–26_ treatment favors the tissue regeneration after skin grafting by avoiding exacerbated inflammation and improving the angiogenesis process.

## Introduction

Skin grafting has been employed to treat several acute and chronic wounds, and the success of the process is depending on an appropriated inflammatory reaction, neovascularization, granulation tissue formation, re-epithelialization, and tissue remodeling (Barrientos et al., 2008). Hence, skin grafting is a highly complex process, mediated by numerous growth factors and inflammatory modulators (Leoni et al., 2015). The healing of wounds begins with clots formation, influx of inflammatory cells, fibroblast proliferation, and capillary damage with subsequent migration and proliferation of endothelial cells to the transplanted area. Then, angiogenesis takes place to deliver nutrients and oxygen to the wound bed, and to improve fibroblast proliferation (Bergers and Benjamin, 2003; Moon and West, 2008; Micallef et al., 2012; Yoo et al., 2013).

Annexin A1 (AnxA1) is a 37 kDa protein positively regulated by glucocorticoids and binds to membrane phospholipids in a calcium-dependent manner (Perretti and D’Acquisto, 2009; Xu et al., 2013). AnxA1 is a member of a family of 13 proteins identified in mammals (Raynal and Pollard, 1994; Hoehenwarter et al., 2008) that contains a small N-terminal region, varying in length and composition, and a central domain consisting of 4–8 repetitions of a highly conserved aminoacid sequence. The N-terminal domain is unique to each member of the annexin superfamily, and it has been extensively shown that this region is responsible for the anti-inflammatory actions of AnxA1 (Perretti and Flower, 2004; Solito et al., 2006; Perretti and D’Acquisto, 2009). In this context, the N-terminal AnxA1 mimetic peptide Ac2–26 (AnxA1_2–26_) inhibits neutrophil migration in inflammatory sites, epithelial cell proliferation, phagocytosis of apoptotic neutrophils by macrophages and neutrophil apoptosis, with especial role on the resolution of the inflammation (Gobbetti and Cooray, 2016). Although AnxA1 is able to interact to membrane phospholipids, the AnxA1 effects are dependent on phosphorylation and interaction with formyl–peptide receptors (FPR), especially FPR2, a G-protein coupled receptor; meanwhile the peptide Ac2–26 is able to interact to both FPR1 and FPR2 (Cooray et al., 2013).

Endogenous AnxA1 is involved in muscle and intestinal epithelial cells repair, as these processes are impaired or delayed in AnxA1 null mice (Leikina et al., 2015; Leoni et al., 2015). Furthermore, the benefits of the use of recombinant AnxA1 or its related peptides, such as AnxA1_2–26_, on mucosal epithelial repair have been fully demonstrated (Leoni and Nusrat, 2016). Hence, along with pro-resolutive actions on inflammation, the beneficial results of AnxA1 or its related peptides on tissue repair have pointed out evidences to therapeutic applications of AnxA1 N-terminal peptides (Gobbetti and Cooray, 2016). However, the role of AnxA1 on skin repair is not well established. Secreted AnxA1 is not essential to skin wound healing, as wound inflammation, closure and the formation of granulation tissue were not altered in AnxA1 null mice (Kreft et al., 2016). Conversely, we recently showed, for the first time, that the systemic pharmacological treatment using AnxA1_2–26_ in mice increased skin allograft survival by inducing the resolution of inflammation, as it caused impaired migration of neutrophils into tissue and enhanced apoptosis of these cells in the site of allograft transplantation (Teixeira et al., 2016).

In order to establish the mechanisms of AnxA1_2–26_ on skin healing process, in this study we investigated the systemic actions of the AnxA1_2–26_ on heterologous skin transplantation in mice, focusing on inflammation and angiogenesis. Altogether, our data suggested that Ac2–26 peptide acts as a facilitating agent in skin allograft transplantation, by limiting local inflammation, inducing fibroblast proliferation and angiogenesis.

## Materials and Methods

### Animals

Male BALB/c wild-type mice, weighing 25–30 g, 6–8 weeks old, were kept on a 12 h light-dark cycle and allowed food and water *ad libitum*. They were anesthetized with ketamine (20 mg/kg) and xylazine solution (2 mg/kg) before each experimental procedure. All experiments were performed according to protocols approved by the Brazilian Society of Science of Laboratory Animals (SBCAL) for proper care and use of experimental animals, and it was approved by the Ethics Committee in Animal Experimentation of São Paulo State University of Sao Jose do Rio Preto (Nos. 074/2013 and 065/2012). The total number of animals used in the *in vivo* experiments was 60.

### Dermis Harvesting and Scaffold Production

Scaffolds were produced at the Northwick Park Institute for Medical Research, London, United Kingdom. Fresh porcine skin was obtained from Large-White/Landrace crossbred pigs after euthanasia. This study was performed according to the regulatory guidelines of the United Kingdom Home Office. Procedures of skin harvesting and scaffold production were described by Mimura et al. (2016). The detailed method is described in the **Supplementary Material**.

### Heterologous Transplantation

To carry out heterologous transplantation we used porcine decellularized skin (scaffolds). Mice were anesthetized, and the surgical procedures were carried out according to Mimura et al. (2016). Details of technical procedures are described in the **Supplementary Material**. Transplanted mice were subjected to daily administration of either PBS or AnxA1_2–26_ (Ac-AMVSEFLKQAWFIENEEQEYVQTVK, Invitrogen, United States) (*n* = 5 animals/group) and sacrificed on days 3, 10, 15, and 60 after transplantation. Pharmacological treatments started 3 days before heterologous skin transplantation. The AnxA1_2–26_ (100 μg/day diluted in sterile PBS) was administrated intraperitoneally (Teixeira et al., 2016).

### Processing of Skin Fragments for Histological Analysis

Samples were fixed for 24 h in 4% paraformaldehyde solution at room temperature. They underwent the process of multiple washes in distilled water, dehydration in graded alcohol, embedding in paraffin wax, sectioning to 5 μm, staining with haematoxylin and eosin (HE) and analyzing on an Axioskop 2-Mot Plus Zeiss microscope (Carl Zeiss, Jena, Germany).

### Quantitative Reverse Transcription Polymerase Chain Reaction (qRT-PCR)

Briefly, total RNA was extracted from the scaffold transplanted area of mice using a commercially available kit (QiagenRNeasy Mini Kit; Qiagen, Hilden, Germany). Tissues were collected from mice subjected to daily administration of either PBS or AnxA1_2–26_ (*n* = 5 animals/group) and sacrificed at 3, 10, 15, and 60 days after transplantation procedure. Pharmacological treatments started 3 days before heterologous skin transplantation. AnxA1_2–26_ (100 μg/day diluted in sterile PBS) was i.p. administrated. Specificities of the technique here employed are described in the **Supplementary Material**.

### Multiplex Assays

Transplanted tissues were macerated in liquid nitrogen and placed in clean, 1.5 mL tubes to which 500 μL of a solution containing protease inhibitor cocktail (GE Healthcare, Amersham, United Kingdom) and Tween 20 (1 μL) (Sigma-Aldrich, Poole, Dorset, United Kingdom) was added to quantify inflammatory mediators interleukin (IL)1β, IL-6, tumor necrosis factor-α (TNF-α), IL-17, and interferon-γ (INF-γ). The description of experimental procedure is described in the **Supplementary Material** and tissues were collected after the treatments described above.

### Dorsal Skinfold Chamber

The dorsal skinfold chamber was implanted in mice under anesthesia, as previously described by Harder et al. (2004). Saline (10 μL) (control), AnxA1_2–26_ peptide (0.4 μg), and/or VEGF-A (10 ng) (*n* = 5 animals/group) were locally applied as previously described by Drewes et al. (2012). Treatments were carried out on the 4th, 5th, and 6th days after chamber implantation. The images obtained before (day 4) and after treatment (day 9) were quantified according to Dellian et al. (1996) and Drewes et al. (2012). The representative scheme of tissue analysis is described in the **Supplementary Material**.

### Cell Culture and Experimental Procedures

Human umbilical vessel endothelial cells (HUVEC) (ATCC-CRL-2873TM) were cultured in 75 cm^2^ plastic culture flasks with DMEM (Invitrogen, Carlsbad, CA, United States) supplemented with 10% fetal bovine serum, L-glutamine (200 mM), streptomycin (0.1 mg/mL) and penicillin (100 U/mL) (Cultilab, Brazil), at 37°C in a humid atmosphere containing 5% CO_2._ Cells were used up to the 3rd passage. HUVEC proliferation, migration and tube formation were performed according to Drewes et al. (2015). The detailed experimental procedures are described in the **Supplementary Material**. HUVECs were seeded (1 × 10^4^ cells/well) and, after cell adhesion, were incubated with PBS (control), AnxA1_2–26_ peptide (30 μM) and/or VEGF-A (10 ng/mL) (Cell Signaling Technology, Danvers, MA, Unites States) for 24, 48, or 72 h to measure proliferation. Semi confluent HUVECs in the matrigel were disrupted with a pipette tip, creating a “groove” in the center of the well. After, the cells gently washed and incubated with PBS (control), AnxA1_2–26_ peptide (1, 10, or 30 μM) and/or VEGF-A (50 ng/mL), for 12 h, to measure the migration into matrigel; 2 × 10^4^ cells/well were incubated with PBS (control), AnxA1_2–26_ peptide (1, 10, or 30 μM) and/or VEGF-A (50 ng/mL) for 2 h and cells were plated under the Matrigel^®^ (Corning, Corning, NY, Unites States) layer to form capillary-like structures for 6 h.

### F-actin Staining by Confocal Microscopy Assay

Human umbilical endothelial cells (1 × 10^4^ cells/well) were seeded on a glass-bottom culture dish and once adhered, they were treated with PBS (control), AnxA1_2–26_ peptide (30 μM) and/or VEGF-A (50 ng/mL) for 2 h. Immediately after the treatment protocol, cells were stained using an F-actin kit (Cytoskeleton, Inc., Denver, CO, United States) and visualized by confocal microscopy (Carl Zeiss LSM 780-NLO, Germany). The description of experimental procedure is described in the **Supplementary Material**.

### F-actin Quantitation With a Fluorescent Plate Reader

HUVECs were cultured to reach 80–90% confluence in 48-well plates. After treatment with PBS (control), AnxA1_2–26_ peptide (30 μM) and/or VEGF-A (50 ng/mL) for 2 h, cells went through the same staining procedure as described above. After being stained, the cells were washed three times with PBS and then 100 μL of wash buffer were added in each well and the fluorescence was read by a Synergy H1 (BioTek^®^, Peru, NY, United States) fluorescent plate reader (excitation wavelength of 535 nm and an emission wavelength of 585 nm). Results are expressed as the median of fluorescence intensity.

### Ultrastructural Immunocytochemical Analysis

To detect the FPR1 (Abcam Cambridge, United Kingdom) and AnxA1 (Zymed Laboratories, Cambridge, United Kingdom), ultrathin sections (∼70 nm) of LR Gold embedded-HUVECs were treated with VEGF-A, AnxA1_2–26_ or both for 2 h and submitted to several steps before being incubated with IgG antibodies conjugated to 20 and 10 nm colloidal gold (British Biocell, United Kingdom). The complete description of the methods is described in the **Supplementary Material**.

### Statistical Analysis

Data were analyzed using Prisma^®^ GraphPad software version 5.00. The results were presented as mean ± standard error of the mean (SEM) and statistical analysis was performed by analysis of variance for multiple comparisons (ANOVA), followed by the Bonferroni adjustment or Student’s t-test. P < 0.05 were considered to indicate statistically significant results.

## Results

### AnxA1_2–26_ Treatment Induces Angiogenesis, Fibroblasts Influx, and Reduces Inflammatory Cytokines Secretion in Transplanted Tissue

**FIGURE 1:**
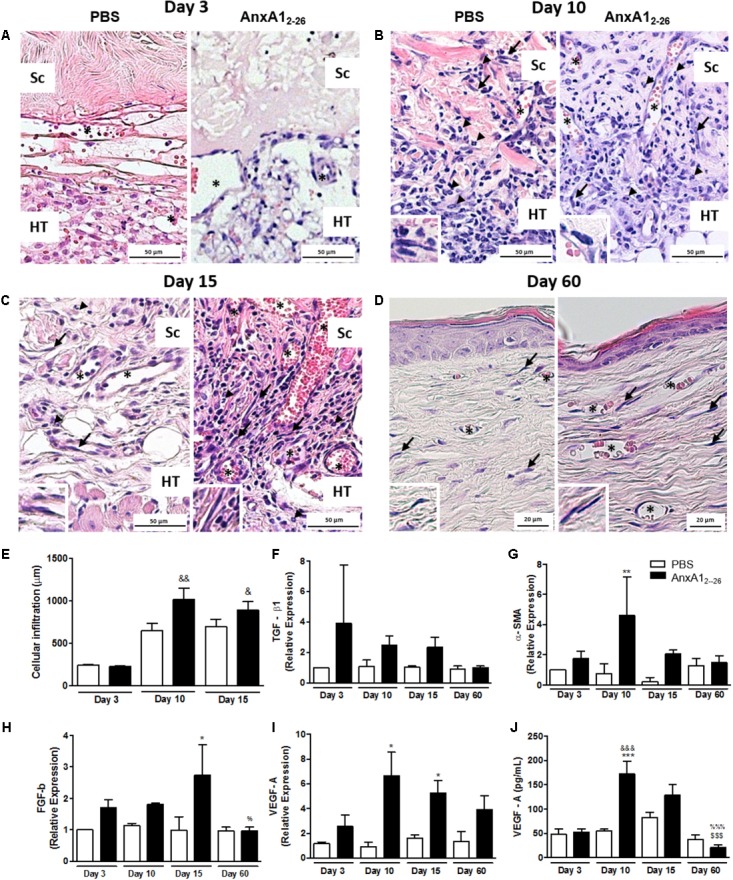
AnxA1_2–26_ treatment improves the heterologous transplantation and induces angiogenesis. Histopathological analyses of skin transplanted fragments without (PBS) and with AnxA1_2–26_ peptide treatment after 3 **(A)**, 10 **(B)**, 15 **(C)**, and 60 **(D)** days post-surgery. Cell infiltration **(E)**, TGF-β **(F)**, α-SMA **(G)**, FGF-b **(H)**, and VEGF-A **(I)** gene expression and VEGF-A protein **(J)** in the transplanted tissue. Host tissue (TH), transplanted scaffold (Sc), vessels (^∗^), fibroblasts (arrows). The inserts show high magnifications of the fibroblasts. The values express the mean ± SEM of five animals per group (ANOVA followed by the Bonferroni’s test). ^∗^*p* < 0.05, ^∗∗^*p* < 0.01, and ^∗∗∗^*p* < 0.001 vs. respective PBS; ^&^*p* < 0.05, ^&&^*p* < 0.01 and ^&&&^*p* < 0.001 vs. day 3; ^$$$^*p* < 0.001 vs. day 10; ^%^*p* < 0.05 and ^%%%^*p* < 0.001 vs. day 15. Staining: hematoxylin & eosin.

**FIGURE 2:**
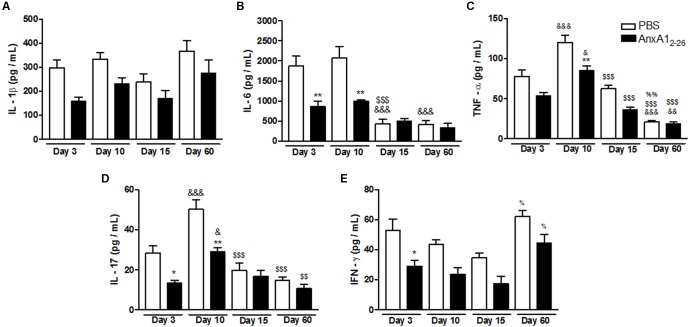
AnxA1_2–26_ treatment reduces the inflammatory response in the heterologous transplantation. Levels of IL-1β **(A)**, IL-6 **(B)**, TNF-α **(C)**, IL-17 **(D)**, and IFN-γ **(E)** in transplanted tissue extracts at days 3, 10, 15, and 60 post-surgery quantified by Multiplex. Data indicate the mean ± SEM of data obtained from five animals per group (ANOVA followed by the Bonferroni’s test). ^∗^*p* < 0.05 and ^∗∗^*p* < 0.01 vs. respective PBS; ^&^*p* < 0.05, ^&&^*p* < 0.01, and ^&&&^*p* < 0.001 vs. day 3; ^$$^*p* < 0.01 and ^$$$^*p* < 0.001 vs. day 10; ^%^*p* < 0.05 and ^%%^*p* < 0.01 vs. day 15.

Systemic treatment of transplanted mice with AnxA1_2–26_ peptide augmented the number of blood vessels on day 10, which was further enhanced up to the 60th day (**Figures 1A–D**). Moreover, AnxA1_2–26_ treatment induced augumentation on the number of vessels and migration of fibroblasts into the scaffold (**Figures 1A–E**), and also enhanced the mRNA levels of mediators involved in the recovery of the transplanted tissue, as detected by greater levels of transforming growth factor-β1 (TGF-β1), myofibroblasts α smooth muscle actin (α-SMA), fibroblast growth factor basic (FGF-β) (**Figures 1F–H**), and mRNA and protein levels of VEGF-A (**Figures 1I,J**). In the inflammatory scenario, we detected reduced levels of the pro-inflammatory mediators IL-1β, IL-6, TNF-α, IL-17, and IFN-γ during the first days after transplantation in the tissue collected from AnxA1_2–26_ treated mice (**Figures 2A–E**).

### AnxA1_2–26_ Treatment Induces *in vivo* Angiogenesis

**FIGURE 3:**
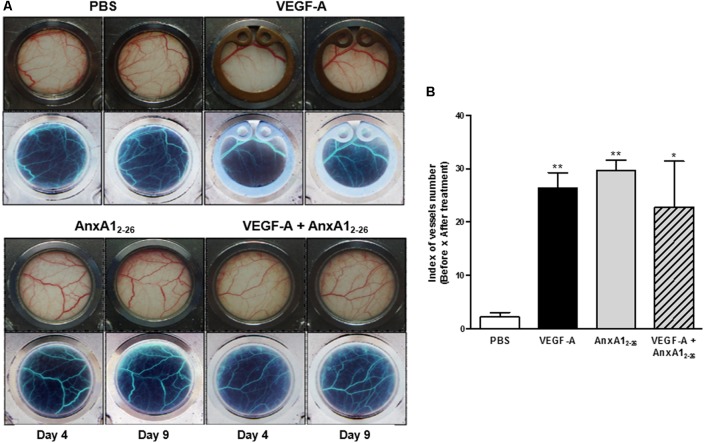
AnxA1_2–26_ increases angiogenesis in a dorsal chamber model in BALB/c mice. Mice were topically treated with Saline (10 μL), AnxA1_2–26_ (1 mg/kg), and/or VEGF-A (10 ng/10 μL) in the dorsal skin. The treatments were administrated once per day, every 2 days, resulting three applications in each mouse. Representative images of the microcirculatory network of dorsal skin were obtained before (day 4) and after (day 9) treatments **(A)**. The images in the upper panel represent the stained normal tissue and in the lower panel, the same computational images obtained after inverting the colors are displayed **(A)**. The quantification of vessels is represented in **B**. The values express the mean ± SEM of five animals per group (ANOVA followed by Bonferroni’s Multiple Comparison Test). ^∗^*p* < 0.05, ^∗∗^*p* < 0.01 vs. PBS.

To assess if AnxA1_2–26_ induces angiogenesis on absence of allograft transplantation, the dorsal skinfold chamber was implanted in mice and topical treatments started 3 days later. This protocol has been employed to avoid interference of surgical stress observed until 48 h of surgery (Drewes et al., 2012). The images obtained showed that local application of VEGF-A, AnxA1_2–26_ or both simultaneously elevated the number of vessels in the dorsal subcutaneous tissue (**Figure 3**). It is noteworthy to mention that angiogenesis induced by VEGF-A treatment was similar in wild type (WT) and AnxA1 null mice (**Supplementary Figure 2**), showing that endogenous AnxA1 is not relevant to the VEGF-A induced angiogenesis in the skin. As previously mentioned, it was recently showed the normal skin repair in AnxA1 null mice (Kreft et al., 2016).

### *In vitro* AnxA1_2–26_ Treatment Induces Endothelial Proliferation and Migration

**FIGURE 4:**
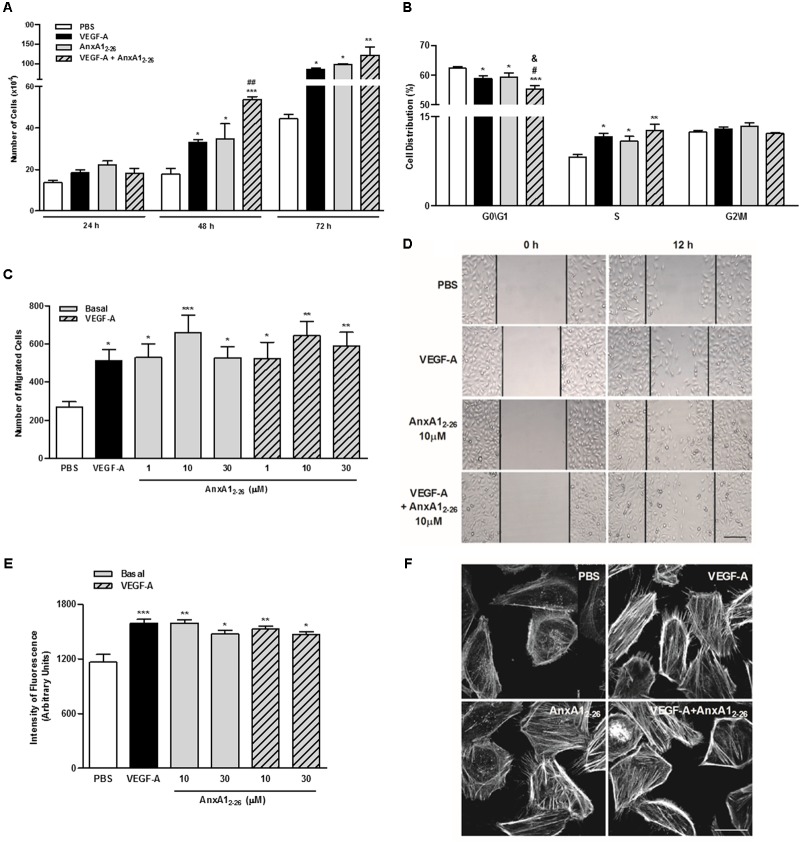
AnxA1_2–26_ increases endothelial cell migration and actin polymerization. HUVECs (1 × 10^4^ cells/well) were incubated with PBS (control), AnxA1_2–26_ (30 μM), and/or VEGF-A (10 or 50 ng/mL) and cell proliferation was evaluated at 24, 48, and 72 h. Results are expressed as the mean ± SEM of cells of two independent experiments in triplicate **(A)**. HUVECs were incubated with different treatments for 48 h, later labeled with PI (50 μg/mL) and the cell cycle phases were evaluated (ANOVA followed by the Tukey’s multiple comparisons test) **(B)**. HUVEC migration was evaluated after 12 h of incubation with PBS (control), AnxA1_2–26_ (1, 10, or 30 μM) and/or VEGF-A (50 ng/mL). Cell migration was monitored with images obtained before (0 h) and after (12 h) treatments **(C,D)**. HUVECs (1 × 10^4^ cells/well) were incubated with different treatments for 2 h and later incubated with rhodamine-phalloidin to evaluate actin polymerization. The intensity of fluorescence was monitored using a fluorescent plate reader **(E)** and by confocal microscopy **(F)**. Scale bar = 10 μm. Results are expressed as the mean ± SEM of cells of two independent experiments in triplicate (ANOVA followed by the Bonferroni’s test). ^∗^*p* < 0.05; ^∗∗^*p* < 0.01 and ^∗∗∗^*p* < 0.001 vs. PBS; ^#^*p* < 0.05 and ^##^*p* < 0.01 vs. VEGF; ^&^*p* < 0.05 vs. AnxA1_2–26_.

As expected, incubation with VEGF-A significantly stimulated the proliferation of HUVECs. Treatment with AnxA1_2–26_ peptide induced cell proliferation and enhanced the effect caused by VEGF-A after 48 h of co-incubation (**Figure 4A**). Moreover, treatment with VEGF-A or AnxA1_2–26_ peptide reduced the percentage of cells in phases G0/G1 and elevated cells in phase S. Cell proliferation and alterations on cell cycle were further augmented by co-treatment with VEGF-A and AnxA1_2–26_ peptide (**Figure 4B**). It is noteworthy to mention that treatments did not induce HUVEC toxicity, measured by the amount of apoptotic and necrotic cells in flow cytometry analysis (**Supplementary Table 1**).

Moreover, AnxA1_2–26_ treatment augmented the migration of HUVECs, similarly, to that evoked by VEGF-A treatment. Furthermore, co-treatment with the peptide and VEGF-A did not cause further migration in comparison with those induced by isolated treatments (**Figures 4C,D**). The fluorescent plate reader and confocal analyses of F-actin, detected by phalloidin binding, showed that all treatments, VEGF-A, AnxA1_2–26_ or VEGF-A plus AnxA1_2–26,_ enhanced actin polymerization (**Figures 4E,F**).

**FIGURE 5:**
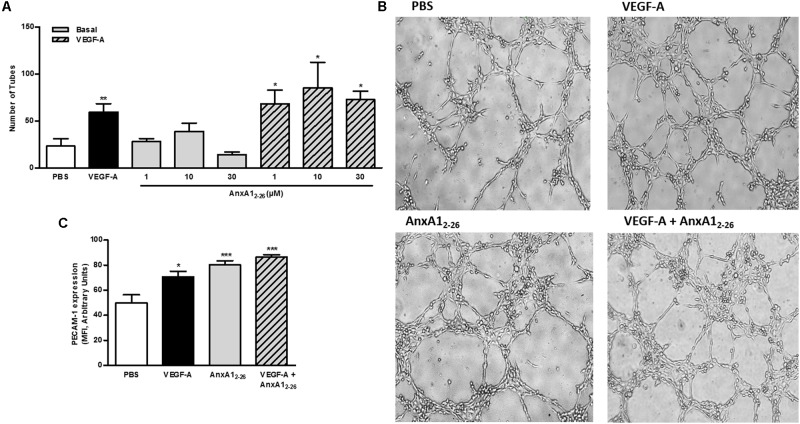
AnxA1_2–26_ does not induce tube formation. HUVECs (2 × 10^4^ cells/well) were incubated with PBS (control), AnxA1_2–26_ (30 μM), and/or VEGF (50 ng/mL) for 6 h on Matrigel^®^and the number of tube structures were quantified using an optical microscope **(A,B)**. PECAM-1 expression was evaluated by flow cytometry **(C)**. Scale bar = 10 μm. Results are expressed as the mean ± SEM of two independent experiments in triplicate (ANOVA followed by the Tukey’s multiple comparisons test). ^∗^*p* < 0.05, ^∗∗^*p* < 0.01; ^∗∗∗^*p* < 0.001 vs. PBS.

Capillary-like tube formation is representative of the latter phase of angiogenesis, as is the primary organization of coalesced endothelial cells. Here we verified that VEGF-A treatment increased the number of tubes, an event not detected in cells treated with only AnxA1_2–26_ peptide. Nevertheless, AnxA1_2–26_ treatment further enhanced the effect produced by VEGF-A (**Figures 5A,B**). The analyses of adhesion molecules involved in homotypic endothelial cell adhesion showed that all treatments, similarly, enhanced PECAM-1 expression on cell membrane (**Figure 5C**).

### VEGF-A or AnxA1_2–26_ Treatments Reduce FPR1 Receptor on Membrane of HUVEC

**FIGURE 6:**
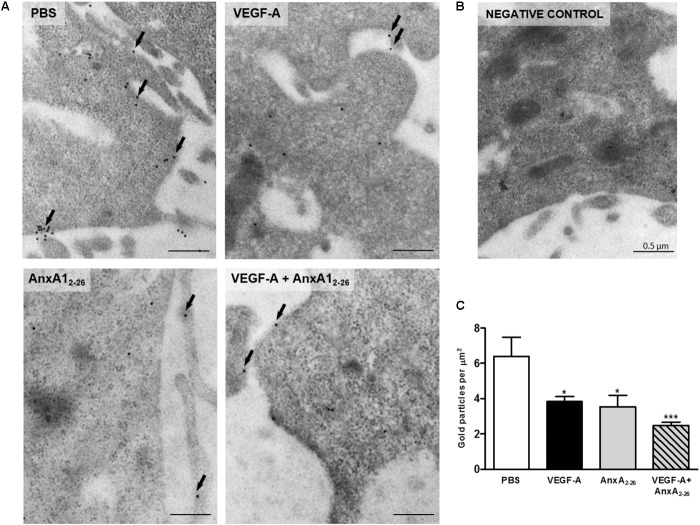
VEGF-A and AnxA1_2–26_ decreases FPR1 expression on HUVECs. FPR1 was detected in the plasma membrane (arrows) and cytoplasm of cells under all experimental conditions **(A)**. Negative control **(B)**. Density of FPR1 immunogold particles in HUVECs **(C)**. Scale bar = 0.5 μm. Data are mean ± SEM of distinct cells analyzed (*n* = 12–20/group) for each condition (ANOVA followed by the Bonferroni’s test). ^∗^*p* < 0.05, ^∗∗∗^*p* < 0.001 vs. PBS.

Images obtained with ultrastructural immunocytochemistry showed that the AnxA1 and FPR1 is downregulated in HUVEC after VEGF-A, AnxA1_2–26_ or VEGF-A plus AnxA1_2–26_ treatments (**Figures 6A,C**). In these cells, gold particles were detected throughout the cytosol, with a significant proportion being observed also in the plasma membrane (**Figure 6A**). No labeling was detected in sections incubated with the control non-immune sheep serum (**Figure 6B**).

## Discussion

In our previous study using a model of mice skin regeneration after decellularized non-crosslinked porcine skin scaffold implantation, we showed that systemic treatment with AnxA1_2–26_ modified the microenvironment of the transplanted area, by limiting the exacerbated inflammatory response at the beginning of the process and favoring angiogenesis and regeneration of the transplanted tissue (Mimura et al., 2016). Furthermore, local application of this peptide evoked angiogenesis in the subcutaneous tissue of mice, and the functional control of the process after treatment seems to be related more prominently to endothelial cell proliferation and migration. However, the ability of AnxA1_2–26_ only to potentiate *in vitro* tubulogenesis caused by VEGF-A and to induce the angiogenesis in *in vivo* conditions, infers that AnxA1_2–26_ may act as a co-adjuvant to growth factors on new vessel formation. Together, our data highlight the mechanisms of AnxA1_2–26_ on skin regeneration and the potential application of the peptide as pharmacological tool.

Previous study of our group had already shown that systemic AnxA1_2–26_ treatment reduced the infiltration of neutrophils into the transplanted tissue and led them to apoptosis (Teixeira et al., 2016), and here we corroborated the anti-inflammatory actions of the peptide by the impaired secretion of pro-inflammatory cytokines at the beginning phase of the tissue regeneration. It is commonly known that acellular skin is not able to promote a specific inflammatory reaction in response to a local graft (Sengor et al., 2005; Ngo et al., 2011), but inflammation occurs due to invasive surgical procedures, and the exacerbated inflammatory reaction impairs tissue regeneration (Diegelmann and Evans, 2004). Since the initial phase, the AnxA1_2–26_ was able to reduce the expression of IL-1β, INF-γ, IL-6, TNF-α, and IL-17, corroborating studies with *in vivo* and *in vitro* models of retina autoimmune disease and uveitis in rodents and human (Girol et al., 2013; Yazid et al., 2015; Cardin et al., 2017). Lima et al. (2017) showed that AnxA1 can acts at multiple regulatory levels to promote resolution of inflammation and may be a common mechanism that account for the pro-resolving actions of pro-resolving molecules.

In the proliferation phase, we have showed that AnxA1_2–26_ treatment enhanced the cellular infiltration of myofibroblasts within the transplanted scaffold, and elevated the levels of regenerative factors, such as FGFs and TGF-β (Mimura et al., 2016). During granulation process, fibroblasts are gradually transformed into myofibroblasts (Hinz, 2016), and they are the predominant mediators of the contractile process (Li et al., 2007). FGFs are growth factor family is also involved in the angiogenesis process, fibroblasts and epithelial cell proliferation, and consequently in the wound healing, allowing for maintenance of transplants (Martin et al., 1992; Lu and Huang, 2013). TGF-β is a growth factor responsible for the differentiation and activation of myofibroblasts (showed by α-SMA-positive cells) (Hinz, 2016; Mimura et al., 2016).

Assuredly, systemic administration of AnxA1_2–26_ enhanced the number of new vessels in the transplanted tissue and enhanced local gene and protein expression of VEGF-A, showing a pro-angiogenic action of the peptide. The direct role of AnxA1 on angiogenesis has not been described, as data regarding AnxA1 on angiogenesis were obtained in *in vivo* and *in vitro* tumorigenesis conditions, and data suggest that the tumor microenvironment is determinant to angiogenic actions of AnxA1 (Oh et al., 2004; Yi and Schnitzer, 2009; Anbalagan et al., 2014). Remarkably, our *in vivo* data showed the ability of AnxA1_2–26_ to induce angiogenesis in the subcutaneous tissue, equivalent to that caused by VEGF-A.

Human umbilical endothelial cell was employed to elucidate the direct actions of Ac_2–26_ on angiogenesis pathways due to well-established data obtained with these cells in the steps of the complex process of vessel formation. We showed here the direct ability of AnxA1_2–26_ to induce cell proliferation, especially accomplished by arresting the cell cycle rather than inhibiting death mechanisms. Furthermore, proliferation and cell arrest were further augmented in AnxA1_2–26_ and VEGF-A co-treated cells, showing synergic effect of AnxA1_2–26_ and VEGF-A on cell cycle phases. AnxA1 actions on cell proliferation are controversial, as both pro- and anti-proliferative actions have been described in different types of cancers (Leoni et al., 2013; Biaoxue et al., 2014; Gastardelo et al., 2014; Liu et al., 2014) and inflammatory cells (Jia et al., 2013). However, our unprecedented data show the direct proliferative role of AnxA1_2–26_ on endothelial cells in the absence of other stimuli.

Furthermore, our results corroborate the actions of AnxA1_2–26_ on cell migration, by acting on the formation of cytoskeletal and protein actin projection at the leading edge of migrating cells, as previously described in muscle, epithelial cells, and fibroblasts (Bizzarro et al., 2012a,b). The pivotal role of AnxA1 in endothelial cell migration had already been shown in VEGF-A induced migration, as the actions of VEGF-A on endothelial cell locomotion depend on the activation of p38/MAP-KAP kinase-2/LIMK1, which phosphorylates endogenous AnxA1, and leads to actin cytoskeletal remodeling (Côté et al., 2010; Pin et al., 2012). However, the direct effect of AnxA1_2–26_ on migration of endothelial cells is shown for the first time in this study.

*In vitro* angiogenesis, quantified by terminal tubulogenesis, was only enhanced by VEGF-A treatment, and AnxA1_2–26_ co-treatment potentiated the VEGF-A effect. The inability of the peptide to evoke the organization of vessels, which depends on a perfect homotypic cell binding, could be unexpectedly, as it has been shown that endogenous AnxA1 mediates the interendothelial cell tight junctions on blood–brain barrier, which stabilizes tight and adherence junctions (Park et al., 2010; Cristante et al., 2013). Moreover, our data showed AnxA1_2–26_ treatment, similarly, to VEGF-A, enhanced the expression of PECAM-1, which is also an important molecule of endothelial cell homotypic interaction (Harris and Nelson, 2010; Park et al., 2015). Hence, it is plausible to suppose that actions AnxA1 and related peptides are different, even though the peptide also binds to FPR1 further than FPR2. Nonetheless, the ability of AnxA1_2–26_ to induce PECAM-1 expression is shown here for the first time, and the molecular basis of this effect will be further investigated regarding the relevance of endothelial PECAM-1 in several pathophysiological conditions, such as cardiovascular and immune diseases (Marelli-Berg et al., 2013; Privratsky and Newman, 2014).

The effects observed here may be dependent on AnxA1_2–26_/FPR1 pathway, as we also showed that endogenous AnxA1, which binds only to FPR2, does not play a pivotal role on *in vivo* angiogenesis induced by VEGF-A. The role of FPR2 on angiogenesis is dual, as serum amyloid A induces functional neovascularization by acting on FPR2 of endothelial cells. Conversely activation of FPR2 on endothelial cells by inflammatory resolving mediators, such as lipoxin A4, reduces the neovascularization on the resolution of inflammation (Prevete et al., 2015). The participation of FPR1 on angiogenesis has been preferentially shown in cancer conditions, and depends on a complex scenario in the tumor microenvironment. Activation of FPR1 on cancer gastric cells reduced the local angiogenesis and cancer growth, depending on pro-resolving mediators of inflammation, such as metabolic activity of lipoxygenases (ALOX5/15) (Prevete et al., 2015, 2017). Differently, the D1 and D2 linear sequences of uroquinase-type plasminogen activator (uPAR) induced angiogenesis by binding to FPR1 on endothelial cells (Prevete et al., 2015), and the blockage of the interaction of uPAR with the receptor that prevents capillary-like tubes formation in co-culture with chondrosarcoma cells (Ingangi et al., 2016). We here showed that VEGF-A or AnxA1_2–26_ treatments down regulated the membrane expression FPR1 in HUVEC, suggesting a direct role of the receptor on angiogenesis process.

Together, data herein presented identified the bioactive AnxA1 derivative peptide AnxA1_2–26_ as a possible therapeutic agent to promote skin regeneration in allograft transplantation, with specific modulation of inflammation and angiogenesis. Moreover, the interrelationship of AnxA1_2–26_ or VEGF-A with FPR1 on endothelial cells opens new pathways to be investigated in order to understand the modulation of angiogenesis process on graft transplantation.

## Author Contributions

JL and CD performed all *in vitro* experiments with HUVEC and intravital microscopy studies, and analyzed the data obtained. JL performed all dorsal skinfold chamber experiments and analyzed the data obtained. KM performed all transplants experiments and analyzed the data obtained. CZ contributed to RNA extraction. CG performed the immune electron microscopy with HUVECs. TA contributed to the production and assessment of the scaffolds. KG performed the production of porcine skin scaffolds and molecular analysis and contributed to the manuscript review. SF and SO supervised the *in vitro* and *in vivo* studies, analyzed the data, and contributed to the writing of the manuscript.

## Conflict of Interest Statement

The authors declare that the research was conducted in the absence of any commercial or financial relationships that could be construed as a potential conflict of interest.
