# Supplementary material for: Annexin A12–26 Treatment Improves Skin Heterologous Transplantation by Modulating Inflammation and Angiogenesis Processes
Source: Front Pharmacol. 2018 Sep 10;9:1015. doi: 10.3389/fphar.2018.01015 (PMC6139386; doi:10.3389/fphar.2018.01015)
Supplement: Supplementary file 1 [file Table_1.pdf]

Supplementary Material

Annexin A1$_{2-26}$ treatment improves skin heterologous transplantation by modulating inflammation and angiogenesis processes

Jéssica Zani Lacerda[1,#], Carine Cristiane Drewes[2,#], Kallyne Kioko Oliveira Mimura[3,#], Caroline de Freitas Zanon[1], Tahera Ansari[4], Cristiane Damas Gil[3], Karin Vicente Greco[4], Sandra Helena Poliselli Farsky[2], Sonia Maria Oliani[1,3,*]

[#] Both authors contributed equally to this research

[1]São Paulo State University (Unesp), Institute of Biosciences, Humanities and Exact Sciences (Ibilce), Campus São José do Rio Preto, São Paulo, Brazil.

[2]Department of Clinical and Toxicological Analysis, Faculty of Pharmaceutical Sciences,University of São Paulo, São Paulo, Brazil.

[3]From the Post-Graduation in Structural and Functional Biology, Federal University of São Paulo, São Paulo, Brazil.

[4]Department of Surgical Research, Northwick Park Institute for Medical Research, University College London, London, United Kingdom.

*** Correspondence:**
Sonia Maria Oliani
sonia.oliani@unesp.br

1 Additional information about the materials and methods

1.1 Dermis harvesting and scaffold production

Scaffolds were produced at the Northwick Park Institute for Medical Research, London, UK. Fresh porcine skin was obtained from Large-White/Landrace crossbred pigs after euthanasia. This study was performed according to the regulatory guidelines of the UK Home Office. Skin was cleansed with soap, shaved and washed with a water and iodine-based solution (10 % w/w Cutaneous Solution - Iodinated Povidone, Videne, Garforth, UK). The skin was dissected from the animal's flank, washed in sterile phosphate buffered saline (PBS, Sigma-Aldrich, Dorset, UK) with an antibiotic/ antimycotic solution (AA; Sigma-Aldrich, Dorset, UK) five times and stored at -20 °C for 24–48 h. The skin samples were defrosted, cut (2 × 2 cm), and subjected to a protocol using a combination of nucleases (RNAse and DNAse) to cleave the sequences of nucleic acids and sodium deoxycholate detergents (SOC; Fluka, Sigma-Aldrich, Dorset, UK), Triton-X-100 (Sigma-Aldrich, Dorset, UK) and a washing buffer containing Tween-20 (Sigma-Aldrich, Dorset, UK) to remove cellular debris from the tissue. The decellularization process was carried out under agitation (Incubator shaker, SciQuip, Shropshire, UK) at 37 °C with 1 % AA (Sigma-Aldrich, Dorset, UK) added to all solutions to minimize bio-burden. Samples were sterilized using 0.1 % peracetic acid (Sigma-Aldrich, Dorset, UK) overnight, under agitation, followed by multiple washes with sterile PBS. Prior to implantation,

all samples were assessed for the presence of cells and heterologous DNA content as described previously (Greco et al., 2015), and results revealed the complete removal of cells and nuclear material from the matrix (data not shown).

1.2 Heterologous transplantation

To carry out heterologous transplantation using skin scaffolds, mice were anesthetized, shaved and disinfected with povidone solution (10 % w/w Cutaneous Solution - Iodinated Povidone, Videne, Garforth, UK), followed by the removal of 1 cm^2 of skin from the dorsum, along the spine of the recipient animals (Teixeira et al., 2016). To help the "taking" of the skin scaffolds, approximately 1 cm^3 of a preparation of collagen paste (Permacol™ paste; Covidien, Leeds, UK) was applied to the wound and the scaffold was immediately placed on the paste and sutured to the native skin (6-0 nylon). Transplanted mice were subjected to daily administration of either PBS (control – TX) or $AnxA1_{2-26}$ (TX + AnxA1) (n = 5 mice/group) and sacrificed at 3, 10, 15 and 60 days after transplantation. Pharmacological treatments started 3 days before heterologous skin transplantation. The $AnxA1_{2-26}$ [(5mg/kg·per day) diluted in sterile PBS] was administrated intraperitoneally (Teixeira et al., 2016).

1.3 Quantitative reverse transcription polymerase chain reaction (qRT-PCR)

Fluorogenic qRT-PCR-based (TaqMan) assay was used to detect amplification of the target genes. Briefly, total RNA was extracted using a commercially available kit (Qiagen RNeasy Mini Kit; Qiagen, Hilden, Germany), according to the manufacturer's instructions, with the following modifications to minimize RNA degradation by abundant skin RNAses. Samples were homogenized using bead-beating technology (Precellys, Bertin Technologies, Montigny-le-Bretonneux, France). Proteins potentially interfering with RNA isolation were removed by incubating the homogenate in 590 µL distilled water and 5 µL Proteinase K solution 20 mg/mL (Life Technologies, Paisley, UK) at 55 °C for 10 min then centrifuged at room temperature for 3 min. Supernatants were combined with 0.5 volumes of ethanol (96-100 %) into a Rnase-Dnase free tube and RNA was isolated through a RNeasy mini column. The concentration and purity of the RNA was analyzed using the Nandrop ND-1000 (NanoDrop Technologies, Wilmington, DE). Complementary DNA (cDNA) was obtained by reverse transcription (RT) of 1 µg of total RNA, with the Superscript III reverse transcriptase system (Invitrogen, Carlsbad, CA, USA) following the manufacturer's protocol and using oligo(dT)15 as a primer. Real-time PCR was performed with the Eco Real-Time PCR System (Illumina, San Diego, CA, USA). The following amplification profile was used: UDG Incubation 50 ^{o}C for 2 min, AmpliTaq Gold 95 ^{o}C for 10 min, PCR 40 Cycles – 95 ^{o}C for 15 sec and 60 ^{o}C for 1 min. For each reaction, a total volume of 20 µL was used, which consisted of 9 µL of diluted cDNA (10 ng/µL of RNA), 10 µL of TaqMan Gene Expression Master Mix (2×) and 1 µL of TaqMan Gene Expression Assay (20×) (Applied Biosystems, CA, USA). Commercially available primers (*GAPDH*, Mm99999915_g1; *VEGFA*, Mm00437306_m1; bFGF Mm01285715_m1; ASMA Mm00725412_s1; TGFb1 Mm01178820_m1 - Applied Biosystems, CA, USA) were used to probe for target mRNA. mRNA data were normalized relative to GAPDH and then used to calculate expression levels. The comparative Ct method was used to measure the gene transcription in samples. Results are expressed as relative units based on calculation of 2-ΔΔCt, which gives the relative amount of target gene normalized to endogenous control (GAPDH) and to the control (sham-operated) samples with the expression set as 1. Negative controls were either RT without enzyme or PCR without cDNA template.

1.4 Multiplex Assays

To quantify the inflammatory mediators IL-1β, IL-6, TNF-α, IL-17 and INF-γ, we used the multiplex instrument LUMINEX xMAP MAGPIX (Millipore Corporation, Billerica, MA, USA). The transplanted tissues were macerated in liquid nitrogen and placed in clean, 1.5 mL tubes to which 500 μL of a solution containing protease inhibitor cocktail (GE Healthcare, Amersham, UK) and Tween 20 (1 μL) (Sigma-Aldrich, Poole, Dorset, UK) was added. The samples were incubated for 1 h at 4 °C under constant agitation and then centrifuged at 21,000 g for 10 min at 4 °C. The protein concentration in the supernatant was measured using a Bradford assay (Biorad, Hemel Hempsted, UK). Antibody beads, controls, wash buffer, serum matrix and standards were prepared following the manufacturer's instructions (MILLIPLEX HCYTOMAG-60K kit). A further 200 μL of wash buffer was added to each well of a magnetic 96-well plate and mixed on a shaker for 10 min. The wash buffer was decanted and 25 μL of standards, controls and samples were added to the wells. Next, 25 μL of assay buffer was added to the samples, and 25 μL of serum matrix was added to the standards. Finally, 25 μL of magnetic beads (coated with a specific capture antibody) was added to all wells and incubated overnight at 4 °C on a shaker. The next day, the plate was washed with wash buffer and incubated with 25 μL of detection antibodies for 1 h on a shaker. Next, 25 μL of streptavidin-phycoerythrin was added to each well and incubated for 30 min on a shaker. The plate was washed and incubated with 150 μL of drive fluid for 5 min on a shaker. Finally, the plate was analyzed using MAGPIX with xPONENT software. Tissues were collected from mice were subjected to daily administration of either PBS or $AnxA1_{2-26}$ (n=5 animals/group) and sacrificed at 3, 10, 15 and 60 days after transplantation procedure. Pharmacological treatments started 3 days before heterologous skin transplantation. The $AnxA1_{2-26}$ (100 μg/day diluted in sterile PBS) was administrated intraperitoneally.

1.5 Flow cytometry

Expression of membrane adhesion molecule platelet endothelial cell adhesion molecule-1 (PECAM-1) was quantified in HUVECs (1×10^5 cells/well) incubated with PBS (control), $AnxA1_{2-26}$ peptide (30 μM) and/or VEGF-A (50 ng/mL) for 6 h, and subsequently incubated with anti-PECAM-1 (1:100, 20 min), and with FITC-goat polyclonal to mouse IgG (1:200, 60 min). Cell cycle was evaluated in HUVECs (1×10^4 cells/well), incubated with PBS (control), $AnxA1_{2-26}$ peptide (30 μM) and/or VEGF-A (50 ng/mL) for 48 h. Afterwards, cells were washed with PBS, trypsinised and fixed by adding cold methanol (75 %) for 1 h. DNA was stained with 200 μL of PI (10 μg/mL) and 20 μL of RNAse (15 μg/mL). The fluorescence was quantified by flow cytometry (FACS Accuri Cytometer; Becton Dickinson, Mountain View, CA, USA) and results were analyzed using the Flow Jo (version 9.1) software. Data from 10,000 cells were obtained and only morphologically viable endothelial cells were considered in the analysis.

1.6 Cell proliferation assay

To investigate the effect of $AnxA1_{2-26}$ peptide on cell growth, HUVECs were plated (1×10^4 cells/well) and, after cell adhesion, were incubated with PBS (control), $AnxA1_{2-26}$ peptide (30 μM) and/or VEGF-A (10 ng/mL) for 24, 48 or 72 h. Later, the number of cells was quantified with Countess® Automated Cell Counter (Invitrogen, UK). To evaluate the analysis of cell proliferation, the counted cells were compared with a growth curve of cells grown without any treatment. The results are expressed as number of cells $\times 10^4$.

1.7 Cell migration assay

Semiconfluent HUVECs were disrupted with a pipette tip, creating a “groove” in the centre of the well (Bürk, 1973). Afterwards, cells were gently washed and incubated with PBS (control), AnxA1_{2-26} peptide (1, 10 or 30 μM) and/or VEGF-A (50 ng/mL) for 12 h. Cell migration was monitored with images obtained before and after treatments, using an Olympus DP73 camera (Olympus, Tokyo, Japan) coupled to a microscope (Olympus, Tokyo, Japan, magnification 50×). The number of cell nuclei that crossed the grove line was determined in three different microscopic fields.

1.8 Tube formation assay on Matrigel®

The tube formation assay was performed on a Matrigel® layer as previously described by Drewes et al. 2015(Drewes et al., 2015). Briefly, 200 μL of Matrigel® were added to each well and incubated at 37 ºC for 1 h to form a gel layer. Subsequently, HUVECs (2×10^4 cells/well) were incubated with PBS (control), AnxA1_{2-26} peptide (1, 10 or 30 μM) and/or VEGF-A (50 ng/mL) for 2 h and cells were plated under the Matrigel® layer to form capillary-like structures for 6 h. Afterwards, the capillary-like structures formed in the gel were photographed (Axioskop II, Carl Zeiss, Germany) and the number of tubules was quantified using Image J software.

1.9 F-actin staining by confocal microscopy assay

HUVECs (1×10^4 cells/well) were plated on a glass-bottom culture dish and once adhered, they were treated with PBS (control), AnxA1_{2-26} peptide (30 μM) and/or VEGF-A (50 ng/mL) for 2 h. Immediately after the treatment protocol, cells were stained using an F-actin kit (Cytoskeleton, Inc, Denver, USA). Briefly, cells were fixed with fixative buffer and permeated with a permeability buffer. F-actin of cells present on glass bottom culture dishes were incubated at room temperature with rhodamine-phalloidin (100 nM) for 30 min. Cells were washed three times with a wash buffer and immediately visualized by confocal microscopy (Carl Zeiss LSM 780-NLO, Germany). Representative photographs were obtained in three different fields using a magnification of 63×.

1.10 Ultrastructural immunocytochemical analysis

To detect the co-localisation of endogenous ANXA1 with the formyl peptide receptor 1 (FPR1), ultrathin sections (~70 nm) of LR Gold embedded-HUVECs were incubated sequentially with the following reagents at room temperature: i) 0.1 mol/L phosphate buffer containing 1 % egg albumin (PBEA); ii) 0.1 mol/L PBS containing 5 % egg albumin (PBEA) for 30 minutes; iii) the sheep polyclonal antibody anti-ANXA1 (1:200 in PBEA) and rabbit polyclonal antibody anti-FPR2 (1:200 in PBEA) (Santa Cruz Biotechnology) for 2 hours, with normal sheep and rabbit sera as controls; and iv) three washes (5 minutes each) in PBEA containing 0.01 % Tween 20. To detect ANXA1 and FPR2, a donkey anti-sheep and goat anti-rabbit IgG antibody (1:50 in PBEA) conjugated to 20 and 10 nm colloidal gold (British Biocell, Cardiff, UK), respectively, were added. After 1 hour, the sections were washed in PBEA, stained with uranyl acetate and lead citrate and examined using a ZEISS EM900 electron microscope. The area of the cell compartment was determined with Axiovision software. The density of ANXA1 expression was showed as the mean ± SEM of immunogold particles per μm^2 from distinct cells analyzed (n=12-20/group).

2 Additional results

2.1 Cytotoxicity analysis of the treatments by flow cytometry

The apoptosis and necrosis rates under different experimental conditions show that the treatments did not induce toxicity in the HUVEC cells.

Supplementary Table 1. AnxA1$_{2\text{-}26}$ treatments do not evoke HUVEC death.

	Cell Viability			Apoptosis			Late Apoptosis			Necrosis		
	**PBS**	**AnxA1$_{2\text{-}26}$ (1 μM)**	**AnxA1$_{2\text{-}26}$ (30 μM)**	**PBS**	**AnxA1$_{2\text{-}26}$ (1 μM)**	**AnxA1$_{2\text{-}26}$ (30 μM)**	**PBS**	**AnxA1$_{2\text{-}26}$ (1 μM)**	**AnxA1$_{2\text{-}26}$ (30 μM)**	**PBS**	**AnxA1$_{2\text{-}26}$ (1 μM)**	**AnxA1$_{2\text{-}26}$ (30 μM)**
**24 h**	90±1	90±2	89±4	5.3±1	3.5±0.4	4.1±1	1.8±0.5	1.8±0.6	2.8±1.2	3.5±0.2	5.2±2.2	4.3±1.5
**48 h**	78±3	76±7	68±2	7.8±3	5.6±0.3	12±4.5	3±0.5	3±0.9	2.5±0.5	10.8±2.3	15.6±6.6	16.9±5.7
**72 h**	83±2	86±0.5	82±1.6	4±0.2	2.8±0.6	3.2±0.3	2.6±0.4	1.9±0.5	2.6±0.2	10.1±1.6	8.9±0.9	12±1.2

3 Supplementary Figures

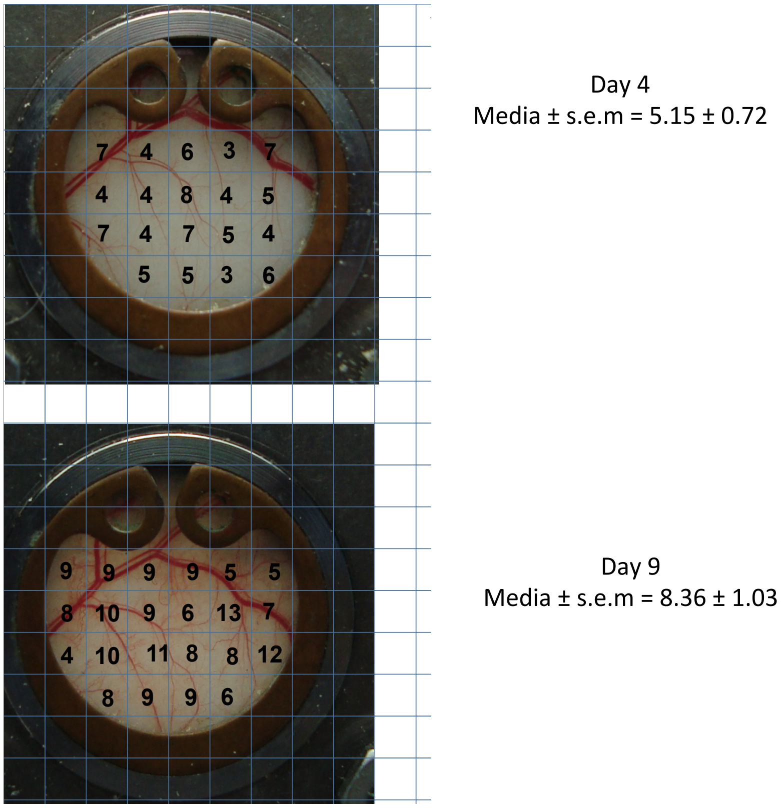

Supplementary Figure 1. Representative image of the quantification of number of vessels in the dorsal skinfold chamber.

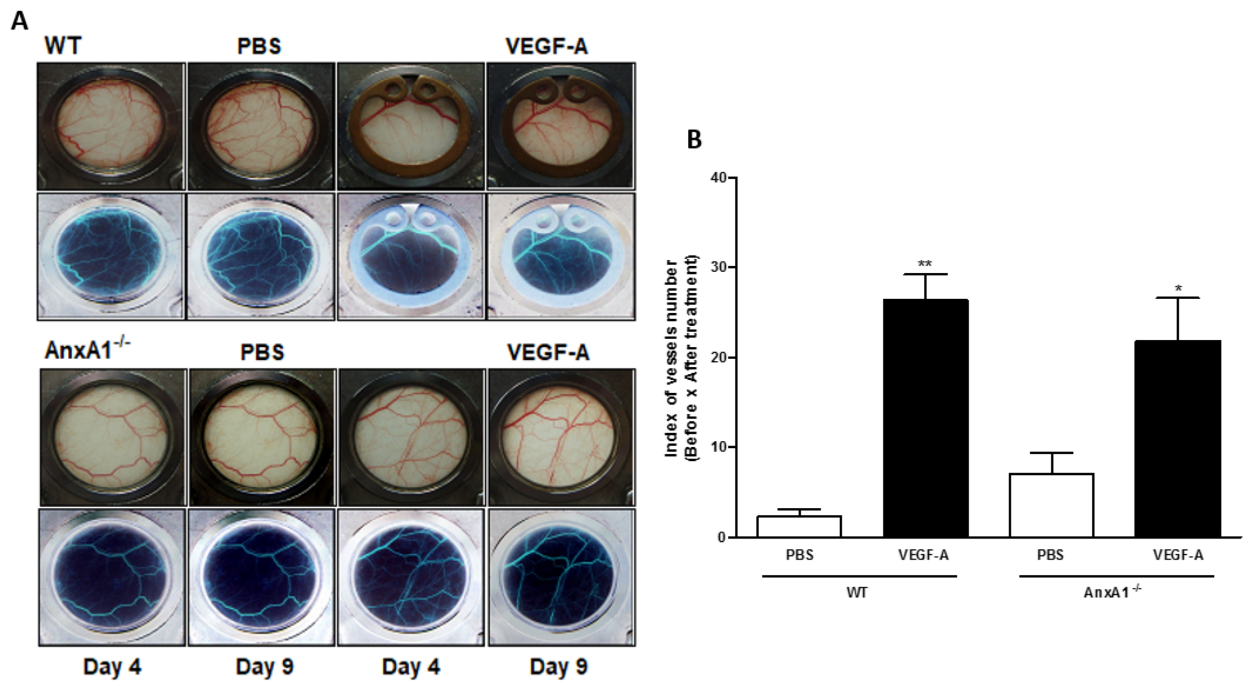

Supplementary Figure 2. VEGF-A induced angiogenesis is not altered in knockout AnxA1 mice (AnxA1-/-). Images obtained showed equivalent number of vessels in AnxA1$^{-/-}$ and WT mice, in both basal and VEGF-A-stimulated animals. Mice were topically treated with PBS (10 µL) or VEGF-A in the dorsal skin. The treatments were administrated once per day, every 2 days, resulting three applications in each mouse. Representative images of the microcirculatory network of dorsal skin were obtained before (day 4) and after (day 9) treatments **(A)**. The images in the upper panel represent the stained normal tissue and in the lower panel, the same computational images obtained after inverting the colours are displayed **(A)**. The quantification of vessels is represented in **B**. The values express the mean ± S.E.M. of five animals per group (ANOVA). $^{*}p<0.05$, $^{**}p<0.01$ vs PBS.

4 References

Bürk, R. R. (1973). A factor from a transformed cell line that affects cell migration. *Proc. Natl. Acad. Sci. U. S. A.* 70, 369–72. Available at: http://www.ncbi.nlm.nih.gov/pubmed/4510281 [Accessed October 21, 2016].

Drewes, C. C., Dias, R. Y., Branco, V. G., Cavalcante, M. F., Souza, J. G., Abdalla, D. S. P., et al. (2015). Post-transcriptional control of Amblyomin-X on secretion of vascular endothelial growth factor and expression of adhesion molecules in endothelial cells. *Toxicon* 101, 1–10. doi:10.1016/j.toxicon.2015.04.002.

Greco, K. V, Francis, L., Somasundaram, M., Greco, G., English, N. R., Roether, J. A., et al. (2015). Characterisation of porcine dermis scaffolds decellularised using a novel non-enzymatic method for biomedical applications. *J. Biomater. Appl.* 30, 239–53. doi:10.1177/0885328215578638.

Teixeira, R. A. P., Mimura, K. K. O., Araujo, L. P., Greco, K. V., and Oliani, S. M. (2016). The essential role of annexin A1 mimetic peptide in the skin allograft survival. *J. Tissue Eng. Regen. Med.* 10, E44-53. doi:10.1002/term.1773.
